# Protein *O*-GlcNAcylation Regulates Innate Immune Cell Function

**DOI:** 10.3389/fimmu.2022.805018

**Published:** 2022-02-03

**Authors:** Hong Dong, Zihao Liu, Haitao Wen

**Affiliations:** ^1^ Department of Microbial Infection and Immunity, Infectious Disease Institute, The Ohio State University, Columbus, OH, United States; ^2^ The Ohio State University Comprehensive Cancer Center, The Ohio State University, Columbus, OH, United States; ^3^ Pelotonia Institute for Immuno-Oncology, The Ohio State University, Columbus, OH, United States

**Keywords:** OGT, O-GlcNAcylation, innate immunity, NF-κB signaling, acute inflammation, antiviral immune response

## Abstract

Metabolite-mediated protein posttranslational modifications (PTM) represent highly evolutionarily conserved mechanisms by which metabolic networks participate in fine-tuning diverse cellular biological activities. Modification of proteins with the metabolite UDP-N-acetylglucosamine (UDP-GlcNAc), known as protein *O*-GlcNAcylation, is one well-defined form of PTM that is catalyzed by a single pair of enzymes, *O*-GlcNAc transferase (OGT) and *O*-GlcNAcase (OGA). Previous studies have discovered critical roles of protein *O*-GlcNAcylation in many fundamental biological activities *via* modifying numerous nuclear and cytoplasmic proteins. A common mechanism by which *O*-GlcNAc affects protein function is through the cross-regulation between protein *O*-GlcNAcylation and phosphorylation. This is of particular importance to innate immune cell functions due to the essential role of protein phosphorylation in regulating many aspects of innate immune signaling. Indeed, as an integral component of cellular metabolic network, profound alteration in protein *O*-GlcNAcylation has been documented following the activation of innate immune cells. Accumulating evidence suggests that *O*-GlcNAcylation of proteins involved in the NF-κB pathway and other inflammation-associated signaling pathways plays an essential role in regulating the functionality of innate immune cells. Here, we summarize recent studies focusing on the role of protein *O*-GlcNAcylation in regulating the NF-κB pathway, other innate immune signaling responses and its disease relevance.

## Introduction

Increased glucose uptake and utilization in immune cells represents a hallmark feature of many inflammatory diseases (1, 2). When immune cells become activated in response to a diverse array of stimuli, glucose serves a major nutrient to fuel increased metabolic demand and support immune cell functions (3–5). Those functions, including phagocytosis, cell migration and cytokine production, etc., are critical for host defense against invading pathogens and tissue injury. After uptake through the glucose transporter, glucose fluxes through three major pathways with distinct destinations and functions, including glycolysis, the pentose phosphate pathway (PPP), and the hexosamine biosynthesis pathway (HBP). The end product of HBP is a monosaccharide uridine diphosphate N-acetylglucosamine (UDP-GlcNAc) (6). The *O*-linked N-acetylglucosamine (*O*-GlcNAc) transferase (OGT) catalyzes the modification of nuclear and cytoplasmic proteins with UDP-GlcNAc on their serine or threonine residue, known as protein *O*-GlcNAcylation (7–10). Meanwhile, *O*-GlcNAcase (OGA) catalyzes the hydrolysis of this sugar modification. Thus, as a dynamic and reversible modification, protein *O*-GlcNAcylation is tightly controlled by this single pair of enzymes OGT and OGA (9, 10).

Since the initial identification of protein *O*-GlcNAcylation in murine lymphocytes by Hart and Torres in 1984 (11), studies over the past four decades have identified numerous proteins as *O*-GlcNAcylation targets. Those proteins, such as kinases, transcription factors, and signaling mediators, are involved in many fundamental biological activities, including gene transcription, protein translation, signal transduction and cell metabolism, etc (6, 12). Since the *O*-GlcNAc moiety can be added to particular serine or threonine residues which act as phosphoryl group acceptor sites, cross-regulation between *O*-GlcNAcylation and phosphorylation has been well documented for many proteins (9). Therefore, it is expected that protein *O*-GlcNAcylation plays a pivotal role in regulating innate immune signaling and inflammatory response through affecting phosphorylation-driving signaling cascades. Indeed, many previous studies have documented the changes in HBP activity and in the level of protein *O*-GlcNAcylation when innate immune cells are activated under inflammatory conditions (13–16). Numerous signaling molecules involved in the NF-κB pathway and other immune pathways have been identified as *O*-GlcNAcylation targets (17–20), highlighting a critical role of protein *O*-GlcNAcylation in modulating innate immune cell function. The underlying mechanisms include, but are not limited to, altered kinase activities, transcription activities and adaptor molecule functions, which are discussed below.

## Protein *O*-GlcNAcylation Regulates NF-κB Signaling

The NF-κB family of transcription factors, including RelA, RelB, c-Rel, NF-κB1 and NF-κB2, plays a central role in regulating functions in all types of immune cells (21). They are central mediators for pro-inflammatory gene expression and inflammatory response (22). Recent studies have provided compelling evidence to suggest an important function of *O*-GlcNAc modification in the regulation of NF-κB signaling.

In line with the notion that glucose metabolism is critical to maintain the transcription activity of NF-κB family members, several previous studies showed that *O*-GlcNAc modification in NF-κB molecules positively regulated their gene transcription functions. One study reported that activation of HBP pathway by either pharmacological (high glucose or glucosamine treatment) or genetic (overexpression of *GFPT* gene encoding glutamine-fructose-6-phosphate transaminase, a rate-limiting enzyme for HBP pathway) strategy resulted in increased expression of NF-κB target genes, which was accompanied with enhanced RelA *O*-GlcNAcylation (23). Yang et al. observed a similarly increased NF-κB activity under hyperglycemic conditions and further identified threonine-322 (T322) and T352 as *O*-GlcNAcylation sites on RelA (19). Genetic mutation of T352 to alanine (T352A) caused a diminished transcriptional activity of RelA due to an increased sequestration of RelA by its inhibitor IκBα. In agreement with the above observation (19), one study using a heterozygous *Oga* gene-deletion (*Oga^+/−^*) mouse model demonstrated that enhanced *O*-GlcNAcylation of RelA on T322 and T352 led to an increased binding of RelA to its target promoter regions, resulting in the hyperactivation of NF-κB signaling and increased cytokine production. This *in vitro* immune phenotype was recapitulated by hyperinflammatory response and exacerbated inflammation-driving tumor growth in the dextran sodium sulfate (DSS)-induced colitis and azoxymethane (AOM)/DSS-induced colitis-associated cancer (CAC) animal models (24). Another study discovered a critical role of RelA *O*-GlcNAcylation in promoting its transcriptional activity with a distinct mechanism. They found that *O*-GlcNAcylation of RelA on T305 was a required step for RelA acetylation on lysine-310 (K310), a necessary modification for its transcriptional activity (20). In addition to immune cells, it was reported that *O*-GlcNAcylation could also promote RelA function in cancer cells. For example, Ma et al. showed that short hairpin RNA (shRNA)-mediated *OGT* gene-knockdown caused a reduced RelA phosphorylation, nuclear translocation, NF-κB transcriptional activity, as well as target gene expression in human pancreatic ductal adenocarcinoma cells (25). These activating effects of OGT-mediated protein *O*-GlcNAcylation on NF-κB signaling were attributed to elevated RelA phosphorylation and acetylation (26). Collectively, those studies suggest a promoting effect of RelA *O*-GlcNAcylation on its transcriptional activity in multiple cell types.

Several NF-κB signaling molecules other than RelA have also been documented to be the targets of *O*-GlcNAc modification with functional consequences. Ramakrishnan et al. discovered *O*-GlcNAcylation of c-Rel on serine-350 (S350) as an important modification to promote its DNA-binding capacity and transcriptional activity (18). The IκB kinase (IKK) is a core enzyme complex of the NF-κB signaling, which is composed of two kinases IKKα and IKKβ and a regulatory scaffolding subunit, IKKγ/NEMO. IKKβ promotes IκBα degradation and induce the activation of NF-κB signaling (21, 27). One study reported that *O*-GlcNAcylation of IKKβ at S733 counteracted its phosphorylation at the same site. Since S733 is an inactivating phosphorylation site (28, 29), *O*-GlcNAcylation of IKKβ at S733 consequently promoted its kinase activity, leading to enhanced NF-κB activity (30). Transforming growth factor (TGF) β-activated kinase 1 (TAK1) is an essential kinase for the generation of inflammatory cytokines in response to the engagement of Toll-like receptors (TLRs), which can form the complex with TGF-β-activated kinase 1 binding protein 1 (TAB1), TAB2 and TAB3 (31). TAK1 activation is regulated by the phosphorylation and ubiquitination of TABs. One recent study revealed that IL-1 and osmotic stress induce the *O*-GlcNAcylation of TAB1 on S395, which in turn promotes TAK1 activation and NF-κB-dependent cytokine release (17). Recently, a whole-body TAB1 knock-in mouse model has been successfully generated in which the single *O*-GlcNAcylation site at TAB1 S393 (mouse S393 corresponding to human S395) was mutated to alanine (*Tab1^S393A^*) (32). No obvious abnormality was observed in homozygous mutant mice. This strain will provide a valuable genetic tool to investigate the function of TAB1 *O*-GlcNAcylation in the regulation of inflammatory response.

## Protein *O*-GlcNAcylation Regulates Acute Inflammation

In addition to promoting proinflammatory TLR-NF-κB signaling, OGT-mediated *O*-GlcNAcylation has been shown to exaggerate inflammatory response by counteracting anti-inflammatory signaling such as STAT3 (signal transducer and activator of transcription 3) signaling in innate immune cells. One study discovered that OGT-mediated *O*-GlcNAcylation of STAT3 at T717 negatively regulates phosphorylation of STAT3 at tyrosine-705 (Y705) (33). STAT3 is a well-established anti-inflammatory factor by promoting the transcription of regulatory cytokines such as IL-10 in myeloid cells (34–36). It was shown that *O*-GlcNAcylation of STAT3 attenuated its phosphorylation and IL-10 production in LPS-challenged macrophages, eventually leading to enhanced cytokine production *in vitro*, as well as exacerbated colonic inflammation and inflammation-driven tumorigenesis *in vivo* (33). *O*-GlcNAcylation of STAT3 is not limited to innate immune cells. Whelan et al. observed that insulin stimulation of adipocytes induced cytosolic translocation of a fraction of nuclear OGT and caused STAT3 *O*-GlcNAcylation (37). Based on the critical functions of STAT3 in regulating multiple pathological processes such as tumorigenesis and insulin resistance, investigation of the functional consequence of STAT3 *O*-GlcNAcylation in non-immune systems may provide novel molecular mechanisms of these diseases and facilitate the development of new therapeutics.

Many effector proteins from one single signaling pathway can be modified simultaneously for any given type of PTM. Therefore, it is not surprising that one PTM such as protein *O*-GlcNAcylation exerts both positive and negative impact on immune signaling at diverse molecular levels. Indeed, several recent studies discovered an anti-inflammatory role of OGT-mediated protein *O*-GlcNAcylation, which is opposite to the observations showing increased NF-κB activation and inflammatory response induced by protein *O*-GlcNAcylation. One study defined a transcriptional repression protein complex containing OGT, the transcriptional corepressor mammalian Sin3A (mSin3A), and histone deacetylase 1 (HDAC1) (38). It was shown that OGT repressed basal and Sp1-driving gene transcription in synergy with mSin3A. In macrophages, LPS stimulation promoted the interaction between OGT and mSin3A and treatment with HBP intermediate metabolite glucosamine attenuated LPS-induced *Nos2* gene expression (39). A follow-up study further characterized that glucosamine-inhibited LPS-NF-κB signaling was dependent on high glucose level in cell culture medium, suggesting that OGT might modulate immune signaling *via* a nutrient-sensing mechanism (40). To examine the overall effect of OGT in the innate immune function, one recent study generated a myeloid-specific *Ogt* gene-deletion mouse strain and observed an inhibitory effect of OGT on innate immune activation through *O*-GlcNAcylation of RIPK3 (receptor-interacting serine/threonine kinase 3). As one of seven members of the RIP serine/threonine kinase family, RIPK3 forms a complex with RIPK1 and plays an essential role in inflammatory cytokine production (41–43) and the execution of necroptosis, an inflammatory form of cell death (41). It was shown that OGT-mediated *O*-GlcNAcylation of RIPK3 at T467 blocked RIPK3-RIPK1 interaction and inhibited downstream immune activation and necroptosis (16). This altered macrophage immune phenotype was recapitulated by an exaggerated inflammatory response in an experimental sepsis model. Another study utilized a hepatocyte-conditional *Ogt* gene-deletion model and revealed OGT as a key suppressor of RIPK3-mediated hepatocyte necroptosis and liver fibrosis (44). Higher protein level of RIPK3 was observed in *Ogt*-deficient hepatocytes, causing excessive necroptosis. *O*-GlcNAcylation of RIPK3 was associated with reduced RIPK3 protein stability (44). Thus, *O*-GlcNAcylation of RIPK3 provides an intrinsic regulatory function to limit excessive inflammation and tissue damage in multiple cell types. Using a high-fat diet (HFD)-induced obesity model, Yang et al. observed an elevated inflammatory cytokine production in *Ogt*-deficient macrophages, which subsequently exacerbates HFD-induced metabolic dysfunctions in liver and muscle (45). Mechanistically, OGT-mediated *O*-GlcNAcylation of ribosomal S6 kinase beta-1 (S6K1) antagonized its phosphorylation and mTORC1 signaling, thus downregulating macrophage inflammation. Collectively, genetic evidence with *Ogt* knockout suggests that OGT-mediated protein *O*-GlcNAcylation negatively regulates myeloid cell immune activation and inflammatory response. Consistent with this concept, several studies observed that administration of glucosamine or OGA inhibitor thiamet G (TMG) reduced the levels of inflammatory cytokines such as IL-6 and TNF-a and improved organ function in multiple inflammation-associated animal models such as sepsis (46–48), trauma-hemorrhage (49) and stroke (50).

**Figure 1:**
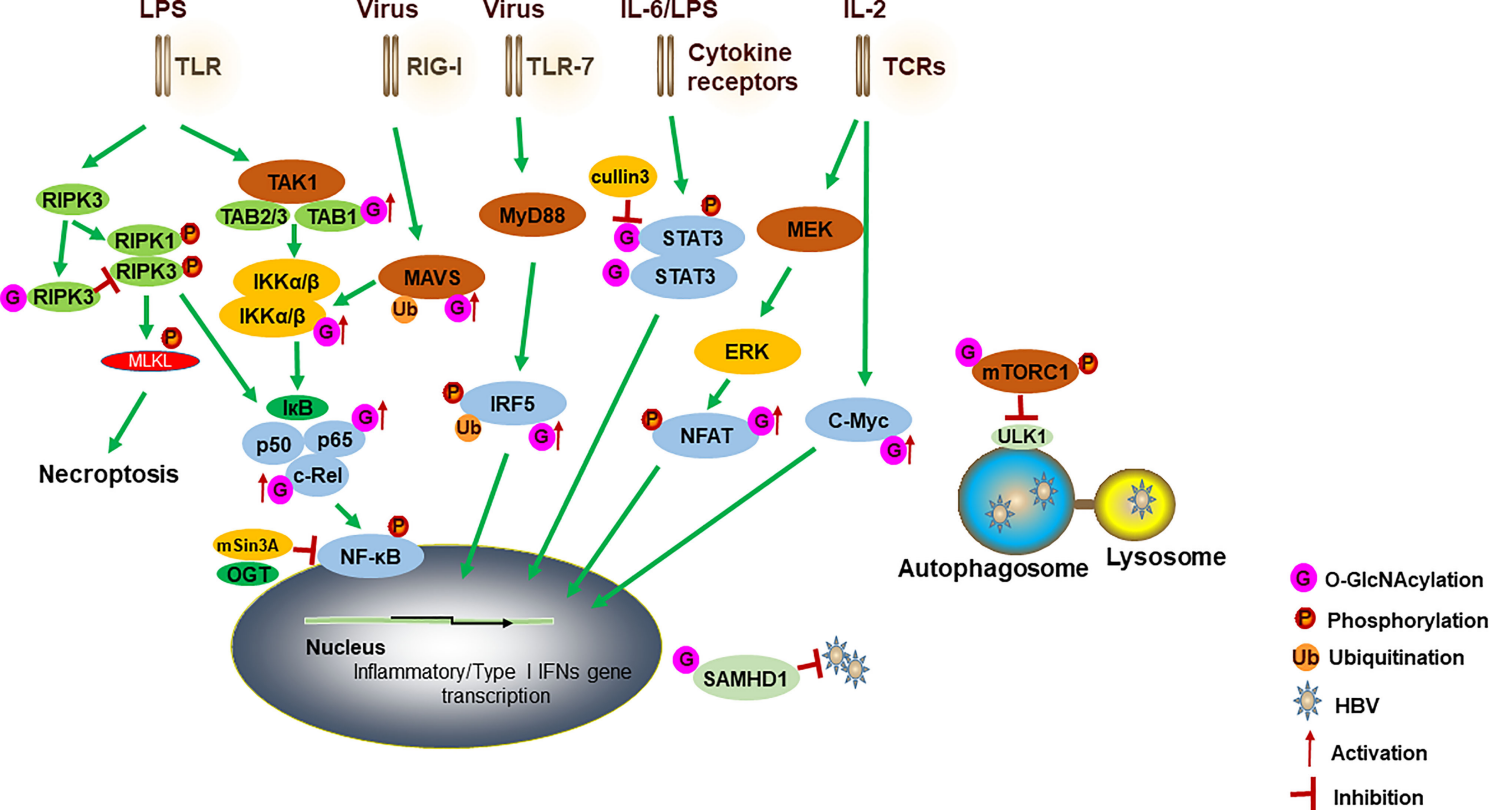
A model for how OGT-mediated protein *O*-GlcNAcylation modulates innate immune cell function. Activation of TLR by LPS induces the aggregation of TAK1, TAB1, TAB2 and TAB3. IKKα and IKKβ complex promotes IκBα degradation and regulates the activation of NF-κB. *O*-GlcNAcylation of TAB1/IKKβ modulates TAK1 activation and promotes IκBα degradation and then results in NF-κB activation and cytokine release. NF-κB subunits (such as RelA and c-Rel) have also been modified with *O*-GlcNAc to regulate their activities. OGT also interacts with mSin3A to inhibit the NF-κB activation. O-GlcNAcylation of RIPK3 inhibits RIPK3-RIPK1 interaction and subsequent necroptosis. A cullin family E3 ubiquitin ligase, cullin 3, inhibits STAT3 *O*-GlcNAcylation and positively regulates STAT3 phosphorylation and its targeted genes. For antiviral innate immunity, OGT-mediated *O*-GlcNAcylation of MAVS promotes its K63-linked ubiquitination, activation of downstream RLR antiviral signaling after VSV infection. *O*-GlcNAcylation of IRF5 is required for its K63-linked ubiquitination and subsequent inflammatory cytokine production after IAV infection. Increased *O*-GlcNAcylation inhibits HBV replication by blocking autophagy initiation through promotion of both mTORC1 signaling and autophagic degradation. OGT-mediated *O*-GlcNAcylation of SAMHD1 promotes its antiviral effect. During the TCR activation and self-renewal, transcription factors NFAT and c-Myc are modified by *O*-GlcNAc to regulate the expression of target genes. TLRs, toll-like receptors; TAK1, transforming growth factor (TGF) β-activated kinase 1; TAB1, TGF-β-activated kinase 1 binding protein 1; IKK, IκB kinase; RIPK3, receptor interacting serine/threonine kinase 3; STAT3, signal transducer and activator of transcription 3; MAVS, mitochondrial antiviral-signaling protein; RLRs, retinoic acid-inducible gene I (RIG-I)-like receptors; VSV, vesicular stomatitis virus; IAV, influenza A virus; HBV, hepatitis B virus; SAMHD1, sterile alpha motif and histidine acid domain-containing protein 1; TCR, T cell receptor.

Genetic studies with the use of *Oga* knockout (24) and *Ogt* knockout (16, 45) models seem to provide contradictory findings. Both the increase and decrease in protein *O*-GlcNAcylation somehow results in a similar hyperinflammatory response. The overall effect of OGT-mediated *O*-GlcNAc signaling in the immune system and inflammation seems to be multifaceted due to targeting a wide range of proteins in different immune signaling pathways (**Figure 1**). When the HBP activity is increased, elevated *O*-GlcNAc signaling promotes activation of the innate immune cells by increasing TLR-NF-κB signaling, as well as counteracting the anti-inflammatory STAT3-IL-10 signaling (33). When HBP activity is decreased, loss of *O*-GlcNAc modification removes the inhibitory mechanisms for proinflammatory mediators such as RIPK3 (16, 44) and mTORC1 (45), and consequently leads to enhanced inflammatory response and organ dysfunction. Therefore, it appears that the loss of homeostasis in the *O*-GlcNAc signaling, instead of a simple one-way increase or decrease, is an important metabolic mechanism to drive the overwhelming immune activation and contribute to the pathogenesis of inflammatory diseases such as sepsis and colitis. It is worth noting that in previous colitis and CAC studies using *Oga* gene-deletion model (24), only cancer cell lines and mouse embryonic fibroblasts (MEFs) with *Oga* gene knockdown or knockout were examined for immune activation, while primary macrophages were not available for tests due to a perinatal lethality of *Oga* whole-body knockout mice. With the recent development of conditional *Oga* gene-deletion model (51) and *Oga* gene knock-in model with null enzymatic activity (D285A) (52), it will be informative to re-examine the function of OGA and its enzyme activity in regulating myeloid cell-mediated inflammatory response.

## Protein *O*-GlcNAcylation Regulates Antiviral Immune Response

Innate immune cells represent the front line of host defense against viral infections (53). During virus infection, cytosolic RNA or DNA species are recognized by retinoic-acid inducible gene I (RIG-I)-like receptors (RLRs) and cyclic GMP-AMP synthase (cGAS), respectively, leading to a robust activation of antiviral immune signaling and upregulation of numerous interferon-stimulated genes (ISGs) in innate immune cells (54–57). Recent studies have discovered an essential function of OGT-mediated protein *O*-GlcNAcylation in promoting antiviral immune responses against both RNA and DNA viruses. One study observed that infection of macrophages with an RNA virus, vesicular stomatitis virus (VSV), caused an elevated HBP activity and protein *O*-GlcNAcylation. Deletion of OGT in macrophages impaired activation of antiviral immune signaling and reduced inflammatory cytokine expression (14). Song et al. observed similar phenotypes of impaired antiviral immune activation in OGT deficient macrophages in response to the challenge with influenza A virus (IAV), Sendai virus and VSV (58). Both studies revealed that OGT-mediated *O*-GlcNAcylation of mitochondrial antiviral-signaling protein (MAVS), a critical adaptor protein for downstream of RLR activation, was a required step for K63-linked MAVS ubiquitination and subsequent activation of antiviral immune signaling. Moreover, myeloid cell-specific deletion of OGT caused an enhanced susceptibility to VSV and IAV challenge *in vivo*, highlighting the importance of OGT-mediated protein *O*-GlcNAcylation in promoting host defense against RNA viruses. Furthermore, Wang et al. reported that deletion of OGT in myeloid cells attenuated IAV-induced cytokine storm and identified *O*-GlcNAcylation of interferon regulatory factor-5 (IRF5) at S430 as an important mechanism promoting the activation of antiviral immune response (59). Using an unbiased microRNA screening strategy, Herzog et al. discovered a potent inhibitory effect of OGT and *O*-GlcNAcylation on the infectivity of hepatitis C virus (HCV), another RNA virus, in human hepatocytes (60). Silencing of *OGT* gene or pharmacological inhibition of OGT led to an enhanced HCV infectivity.

In addition to antiviral effects against RNA viruses, two recent studies also reported the antiviral function of *O*-GlcNAc signaling against a DNA virus, hepatitis B virus (HBV) (61, 62). HBV is a major human pathogen, causing the development of chronic liver diseases, cirrhosis and hepatocellular carcinoma (HCC) (63). Wang et al. reported that genetic or pharmacological inhibition of OGT increased HBV replication and hepatitis B surface antigen (HBsAg) production. This effect was due to the blockade in the autophagosome-lysosome fusion step when *O*-GlcNAc signaling was inhibited, thus causing diminished autophagic degradation of HBV virions and proteins (61). The critical role of OGT in driving autophagic flux has also been recently documented in hepatocytes without virus infection (64), suggesting a general requirement for *O*-GlcNAc signaling to maintain autophagic activity. Another study observed a similar phenotype of increased HBV replication when OGT was inhibited with a distinct mechanism. It was shown that OGT-mediated *O*-GlcNAcylation of sterile alpha motif and histidine acid domain-containing protein 1 (SAMHD1) played a critical role in promoting antiviral effects (62). SAMHD1 is a deoxynucleotide triphosphate triphosphohydrolase (dNTPase) and blocks viral DNA synthesis by reducing intracellular dNTP pools. Therefore, SAMHD1 has been demonstrated as a critical restriction factor limiting the replication of retroviruses and certain DNA viruses including HBV (55). Wang et al. revealed that *O*-GlcNAcylation of SAMHD1 at S93 enhanced SAMHD1 protein stability, thus improving its antiviral activity. Collectively, recent studies provide strong genetic evidence supporting OGT-mediated protein *O*-GlcNAcylation as a critical host defense mechanism linking cellular metabolism to antiviral immunity (**Figure 1**).

Since OGT-mediated protein *O*-GlcNAcylation provides antiviral benefit against both RNA and DNA viruses, a possibility is raised that protein *O*-GlcNAcylation may function through some unified cellular mechanism(s) to restrict virus replication. It has been well recognized that profound metabolic changes occur during viral infection, leading to an increased nutrient availability and permissive intracellular environment that are beneficial for viruses to accomplish their life cycles. For example, viral infection reprograms lipid metabolism in host cells towards an enhanced activity of the fatty acid biosynthesis pathway, causing increased accumulation of neutral lipid species in lipid droplets (LDs) (65–68). This enhanced fatty acid and LD biosynthesis is required for virus replication due to the increased availability of free fatty acids for virus membrane assembly, as well as generating membrane platform for virus genome replication (69). As a result, pharmacological or genetic inhibition of the fatty acid biosynthesis pathway and LD formation have been shown to effectively inhibit virus replication across a wide range of virus types (65, 70–72), including SARS-CoV-2 (73). It has been known for a long time that *O*-GlcNAc signaling exerts a significant impact on lipid metabolism (8, 74, 75). One more recent study characterized *O*-GlcNAcylation of the TATA-box binding protein (TBP) as an important mechanism affecting the transcription of lipid metabolic enzymes (76). Deletion of *O*-GlcNAcylation of TBP on T114 resulted in a significant lipid metabolism reprogramming towards enhanced LD formation. Whether or not OGT/*O*-GlcNAc signaling-mediated antiviral response involves lipid metabolism reprogramming requires further investigation.

Apart from antiviral innate immunity, the CD8^+^ T cell-mediated immune response is another key host immune strategy to eliminate virus in a more specific and efficient manner. Of the two lymphocytes that carry out adaptive immunity, T cells function as a versatile “player” in multiple aspects, including pathogens killing, immunoregulation, and homeostasis. Inevitably, such intensive immune activities are associated with increased glucose consumption and protein modification. UDP-GlcNAc has been reported to participate in T cell activation, self-renewal and immunosuppression through OGT-mediated protein *O*-GlcNAcylation (77, 78). Emerging evidence has identified the extensive crosstalk of *O*-GlcNAcylation with phosphorylation on a wide range of signaling molecules in T cells (79). OGT-mediated protein *O*-GlcNAcylation is necessary for T cell bioactivities (13).

In T cell, recognition of specific antigens *via* T cell receptors (TCR) elicits the initiate signal for T cell activation. Following the activation of TCR, multiple transcription factors, including NF-κB and nuclear factor of activated T cells (NFAT), are activated to regulate the expression of activation-associated genes. Golks et al. have demonstrated that NFAT and NF-κB were modified by *O*-GlcNAc during T cell activation (77). OGT deficiency significantly impairs TCR-induced T cell activation and inflammatory cytokine production (77). This finding was consistent by another study using OGT inhibitor to treat human T cells, resulting in decreased IL-2 production upon TCR stimulation (15). Due to the dynamic and reversible feature of protein *O*-GlcNAcylation, OGT-mediated protein modification enables a T cell to adjust its activation process in response to various pathogens.

It is increasingly recognized that OGT is involved in various T cell activities. Apart from T cell activation and immunomodulation, OGT-mediated protein *O*-GlcNAcylation was also demonstrated to promote T cell self-renewal through interaction with Notch signaling and metabolism in thymocytes *via O*-GlcNAcylated c-Myc (78). Ablation of *O*-GlcNAc signaling disrupts T cell development and leads to T cell apoptosis and malignant transformation (80). Thus, the importance of OGT in T cells is to link the metabolic processes with immune activities, facilitating T cells to integrate these multi-aspects signals in response to different stimuli (**Figure 1**).

## Conclusions

A wealth of evidence therefore points to an intimate relationship between protein *O*-GlcNAcylation, activation of immune signaling and inflammatory responses. This occurs through the *O*-GlcNAcylation of multiple effector proteins, such as kinases, transcription factors, and metabolic enzymes, in response to a diverse range of stimuli. Both positive and negative influences of the *O*-GlcNAc signaling on immune activation and host defense response have been characterized, which reflects the complexity of this PTM in fine-turning immune signaling networks. Significant progress has been recently achieved on the development of small-molecule compounds targeting the enzymatic activity of OGT (81, 82). The hope is that the recent insights into the molecular basis of how protein *O*-GlcNAcylation affects immune signaling will help in the design of new therapies.

## Author Contributions

HD and ZL wrote the first draft of the manuscript, conducted the literature search and contributed to the final draft. HW revised and wrote the final draft. All authors have contribution to the article and approved it for publication.

## Funding

This work was supported by National Institutes of Health (NIH) grants R01GM120496 and R01GM135234 (HW).

## Conflict of Interest

The authors declare that the research was conducted in the absence of any commercial or financial relationships that could be construed as a potential conflict of interest.

## Publisher’s Note

All claims expressed in this article are solely those of the authors and do not necessarily represent those of their affiliated organizations, or those of the publisher, the editors and the reviewers. Any product that may be evaluated in this article, or claim that may be made by its manufacturer, is not guaranteed or endorsed by the publisher.
